# Backcountry Travel Emergencies in Arctic Canada: A Pilot Study in Public Health Surveillance

**DOI:** 10.3390/ijerph13030276

**Published:** 2016-03-03

**Authors:** Stephanie K. Young, Taha B. Tabish, Nathaniel J. Pollock, T. Kue Young

**Affiliations:** 1 Institute for Circumpolar Health Research, Yellowknife, NT X1A 3X7, Canada; stephanie.young@ichr.ca; 2 Qaujigiartiit Health Research Centre, Iqaluit, NU X0A 0H0, Canada; taha.tabish@qhrc.ca; 3 Labrador Institute, Memorial University, Happy Valley-Goose Bay, NL A0P 1E0, Canada; 4 School of Public Health, University of Alberta, Edmonton, AB T6G 1C9, Canada; kue.young@ualberta.ca

**Keywords:** Canada, arctic regions, Indigenous, aboriginal, rural health, search and rescue, transportation

## Abstract

Residents in the Canadian Arctic regularly travel in remote, backcountry areas. This can pose risks for injuries and death, and create challenges for emergency responders and health systems. We aimed to describe the extent and characteristics of media-reported backcountry travel emergencies in two Northern Canadian territories (Nunavut and Northwest Territories). A case-series of all known incidents between 2004 and 2013 was established by identifying events in an online search of two media outlets, Nunatsiaq News and Northern News Services. We identified 121 incidents; these most commonly involved young men, and death occurred in just over 25% of cases. The territories differed in the seasonal patterns. News media provides a partial source of data to estimate the extent and characteristics of backcountry emergencies. This information is needed to improve emergency preparedness and health system responsiveness in the Arctic.

## 1. Introduction

Travel in the backcountry is a common and necessary part of life for residents in the Canadian Arctic. In this region, travel in remote areas is commonly referred to as being *on the land*, and related activities often carry cultural and spiritual importance, in addition to being a required aspect of northern transportation. Although many people travel and spend time on the land safely, structural and environmental factors create unique risks for transportation-related emergencies, which can result in use of emergency health care services, injuries, and death. Monitoring backcountry emergency events in the Arctic is an essential component of community safety planning, emergency preparedness, and health care delivery. 

In Canada, the two northern territories of Nunavut and the Northwest Territories (NWT) occupy about one-third of the nation’s land mass, with a combined population of only 73,400 [1]. These territories are home to the Indigenous Inuit and Dene people, who account for 85% of the population of Nunavut, and 50% of the population of NWT [2]. Vehicle traffic within communities in these territories is common; although it is limited beyond municipal boundaries, travel is necessary for employment, recreation, and accessing services such as medical care. Seasonally, people travel by snowmobile, all-terrain vehicle, boat, and by foot year round. Backcountry travel networks expand during winter and spring months via winter roads and trails, which allows for easier access to the backcountry and other communities.

Among Indigenous populations, knowledge and skills related to on-the-land travel is a historically entrenched part of community life, and has an integral role in personal and community wellbeing [3,4]. For example, subsistence hunting and fishing is common, as is berry picking; together these are essential aspects of local food security, and they usually require travel outside of communities [3]. Spending time on the land is also an important aspect of culture, and many families travel to seasonal camps or cabins [3]. 

The rapid rate of climatic and environmental changes in the Arctic affects local ecosystems and Indigenous peoples’ ability to travel on the land [3,5]. Across the Canadian Arctic, melting permafrost, permafrost erosion, less predictable ice conditions, increased forest fires, unpredictable weather patterns, and changing wildlife habitats are expected to increase the safety risks to travelers in the backcountry [5]. 

Globally, transportation injuries are an important cause of morbidity, disability, and mortality [6]. However, evidence suggests that the problem is considerably worse for Indigenous people, especially those in remote, northern communities where extreme cold weather and cold water temperatures are common [7,8]. In a rural environment, the increased duration between dispatching a rescue team, extracting the victims, and arriving at a medical facility can be expected to elevate the risk for severe health complications and death.

Most northern communities have access to a combination of formal and informal systems to respond to missing persons, accidents, or other backcountry emergencies. Arctic land and marine search and rescue (SAR) is provided through partnerships between the police, military, coast guard, and volunteer SAR organizations. Some communities have developed local teams to complement established systems, or as alternatives, and have also worked to improve first responder skills among community members [9]. The multiplicity of organizations highlight the lack of a centralized and standardized database of SAR activities across Canada [10].

## 2. Materials and Methods 

In this study, we aimed to describe the extent and characteristics of backcountry travel emergencies and their health consequences using news media sources. Previous studies have explored the use of news media as components of injury surveillance [11,12]. This study is part of a broader inquiry on search and rescue events that will capture data from medical and coroner records, and key informant interviews. We developed this pilot study to assess the utility of media reports as a complementary data source. We sought to answer the following questions:

1. How many backcountry emergency events in Nunavut and the NWT did news media outlets report from 2004 to 2013?
2. What were the most common environmental conditions, methods of transportation, and primary causes of backcountry emergency events?
3. What were the common outcomes reported?

We established a case series by conducting an online search for news stories reported in two media outlets in Northern Canada—*Nunatsiaq News* [13] and *Northern News Services* [14]. We identified cases from the online archive for each publication using the search terms “emergency,” “rescue,” “missing,” and “search.” During an exploratory search, we also used terms including “tragedy” and “drowning” among others; however, we excluded them from our final search strategy because they lacked specificity or did not increase the number of search results.

An event was included if it: (1) occurred in the backcountry, or outside of communities, in Nunavut or the NWT; (2) resulted in an emergency, *i.e.*, medical emergency, mechanical problem, missing person(s), or death; (3) occurred between 1 January 2004 to 31 December 2013; and (4) was a unique case. For events with multiple articles written about them, all articles were used to extract event-related data. We excluded aeronautical events. Two investigators independently reviewed the search results from each media outlet to determine eligibility of cases. The authors met to discuss unclear cases and resolve disagreements. We developed a standardized data collection form to ensure consistent extraction. 

We cross-checked our database for duplicate events, and extracted event-related data from the eligible articles including demographics, reason or cause of emergency, purpose of travel, and other details. Where possible, we extracted the primary reason for travelling in the backcountry including tourism and hunting/harvesting. We coded the reason as “travel” when transportation between two locations was the main purpose of the outing; backcountry travel is often necessary to access services or retail, or visit family in other communities. We used descriptive statistics to examine demographic, environmental, and health-related trends. We generated a density map depicting the travelers’ community of origin using *Mapsdata* [15]. We used a public domain data source; therefore, this study did not require approval by a research ethics board. 

## 3. Results

**Figure 1:**
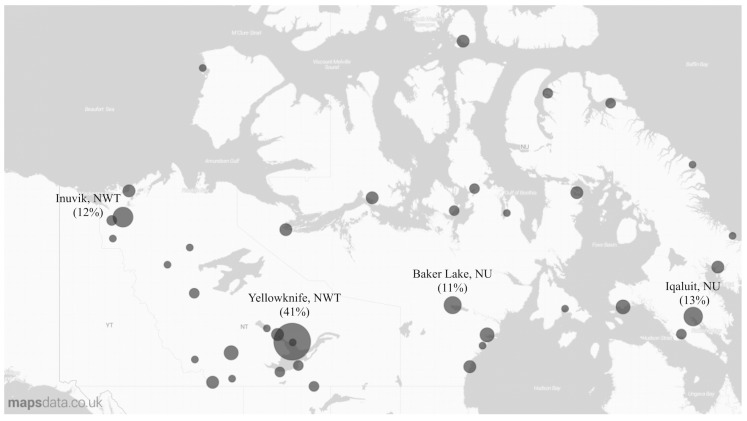
% of territorial events by community of origin.

We identified 55 backcountry travel emergencies in Nunavut involving 159 individuals and 66 emergencies in the NWT involving 129 individuals from 2004 to 2013. Gender information was available in 86% of cases (*n* = 105), the overwhelming majority of whom were male (86% of individuals in Nunavut and 78% in the NWT). Emergency events occurred more often among younger individuals, especially those aged 16 to 25 years. SAR organizations were involved in 74% of cases in both territories. The mean number of individuals in a party was 2.9 in Nunavut and 2.7 in NWT, though the number ranged from solo travellers up to groups as large as 34. The majority of travellers originated in the larger communities including Yellowknife, NWT, and Iqaluit, Nunavut (Figure 1). 

**Figure 2:**
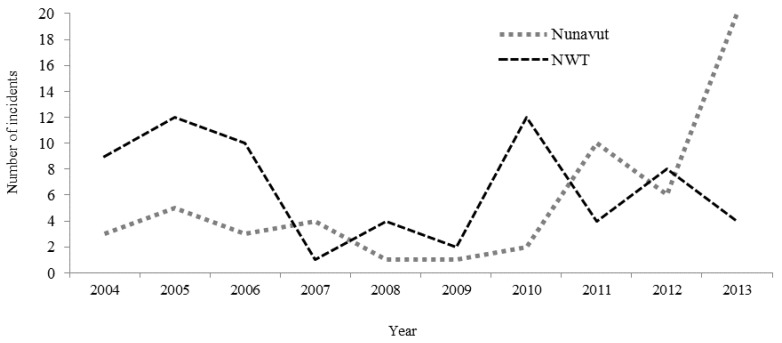
Number of events by year and territory.

Both territories experienced substantial variation in the number of annual events (Figure 2). Averaged over the 10-year period, the annual rate of incidents was 17.2/100,000 in Nunavut and 15.9/100,000 in the NWT, or 16.5/100,000 in the two territories combined. Events occurred most often in November/December in Nunavut and in July/August in the NWT. 

**Figure 3:**
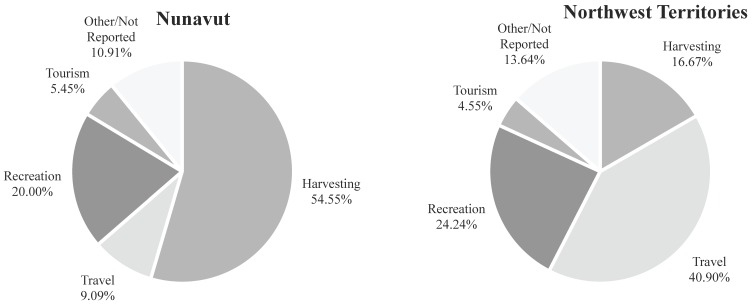
Reasons for being in the backcountry.

The two most commonly reported reasons for being in the backcountry were harvesting (hunting for food) in Nunavut, and transportation/travel in the NWT (Figure 3). Overall, most incidents occurred on land (43%), with fewer on water (35%) or ice (22%). Environmental causes related to ice and water conditions were the most frequently reported contributing factor for events in the NWT. These included falling through the ice, ice jams, fast currents, hitting a sandbar, boat capsizing, and falling into the water. In Nunavut, weather conditions, including high winds, low visibility, and blizzards, were the most frequently reported primary cause of the emergency event. Other commonly reported precipitants in both regions included mechanical problems and collisions. In one case, an individual left the community in response to symptoms related to mental illness; this then became a backcountry emergency event. In the NWT, boating was the most common mode of transportation involved in an incident (35%), followed by snowmobiles (24%), other vehicles (17%), and on foot (14%), with 11% unknown. In Nunavut, snowmobiling was the most common mode of transportation involved in an incident (44%), followed by boats (28%) and ATVs (11%), with 13% unknown. 

In general, articles did not contain reliable information about health status or primary medical problems experienced by individuals. However, some information about the outcome of each emergency event was available, whether individuals survived or not, for 89% (*n* = 49) of cases in Nunavut and 80% (*n* = 53) of cases in NWT. Among those with definitive outcome information, 23% of cases (*n* = 28) resulted in the death of at least one member of the travel party and 60% of cases (*n* = 73) resulted in all members of the travel party being found alive. Individuals were still missing as of the latest media reports of an incident in 17% of cases in Nunavut and the NWT combined. A limited number of articles reported non-fatal health status related to the event, which included hypothermia, fractures, and dehydration. 

## 4. Discussion

This analysis of media-reported backcountry emergency incidents helps us to understand the extent and characteristics of travel-related emergencies that have occurred in Northern Canada. Emergency preparedness in the Arctic is attracting significant attention, and communities are currently aiming to improve their emergency preparedness capacity. A region-wide review has not been undertaken before, and nationally there is no published data on the extent and characteristics of backcountry emergencies [10], although data is available for other circumpolar and remote contexts [12,16]. This pilot study is a first step in a comprehensive assessment of the extent and characteristics of backcountry emergencies.

We do not have comparable annual rates of the occurrence of backcountry incidents. The published rate of 1900/100,000 in one Labrador Inuit community [8] far exceeds our rates of 16.5/100,000, and is alarmingly high. However, since that one community had a small population (<1200), the rate, which was based on two years of data, can be expected to be highly unstable. An Alaskan study of falling-through-ice incidents reported annual rates that ranged from 2 to 4.7 per 100,000, though the rate for Alaska Natives was nearly twice as high (risk ratio: 1.7) [12].

The age-sex distribution of the individuals involved in these incidents identifies young men as the group at highest risk, which is similar to a previous study [12]. That events occurred most often in the winter months (November/December) in Nunavut corresponds with the shoulder months of sea ice formation. In the NWT, the highest number of incidents occurred during the summer months (July/August), which are the months when boating is most frequent. This difference can be attributed to the fact that all but one community in Nunavut are located on the sea coast, whereas the great majority of communities in NWT are located inland on the shores of rivers and lakes. We also observed differences between the territories with regard to the reason for being on the land, environment of incident, and reported cause. These differences suggest the variety of community experiences across Northern Canada, and the necessity for regional- and community-specific emergency preparedness plans.

As a pilot study, several limitations were evident. It is likely that we did not capture all backcountry travel emergencies, and that death-related incidents may be overrepresented. Underreporting may be due to limited news coverage in some communities. Additionally, emergencies that do not require search and rescue support, or those in which individuals do not seek medical attention, may not be officially reported. For the events we did identify, information was often incomplete and inconsistently reported, especially related to health outcomes and Indigenous identity. Factors such as use of alcohol or drugs were rarely reported, despite their roles as an important risk factor for injuries. However, the lack of reporting on alcohol use is consistent with a previous newspaper study [12]. While we collected data from two major Northern news sources, additional emergency events may have been reported by other news outlets. Since events were reported and published as they unfolded in real time, a single incident may have multiple articles. Moreover, we were able to aggregate information from sequential reports.

In public health surveillance, not being able to capture the “true” number of health events for a disease or health condition is not a serious disadvantage, if the reason for under-reporting is consistent over time. For example, pneumonia deaths (at one extreme) and the purchase of cold medicines (at the other extreme) are often used to track the progress of influenza epidemics, even when neither reflects the true number of cases. In our study, describing the pattern of reported events can be helpful in developing prevention programs. 

Overall, we found that the news media is a timely, free, and accessible data source, though it likely over-represents extreme cases, and incidents close to communities with media reporters. Additionally, there is no standard format on how events were reported. As with other secondary data, the primary collector—in this case the reporter—is mainly interested in attracting the attention of the reader, not data consistency, comprehensiveness, or validity. Nevertheless, internet-based media reports are increasingly used as a source of public health surveillance data, for example in infectious disease outbreak control [17]. They also may capture contextual details that are not commonly part of administrative health databases [11]. When additional, but incomplete, sources of data are available, such as police, medical, and coroner’s records, the capture-recapture method can be used to provide an estimate of the “true” prevalence of the condition of interest [18]. Review of these quantitative data, together with qualitative interviews of key informants in the communities, is the next step in our team’s research. By combining multiple data sources, our aim is to gain a more complete understanding of factors that influence local risks, and community health and emergency response efforts.

The formal health care system tends to focus on patients who present at a facility to obtain care from providers. Less attention is directed at community members who spend a portion of their daily life away from the community and are exposed to emergencies that may necessitate access to medical care from afar. This is not a major issue in urban areas, but is a fact of life in the Arctic. Agencies responsible for retrieving such patients generally are not part of the formal health care system, and fall outside the purview of health system planning. Health service providers and health policy makers respond in an *ad hoc* manner to backcountry emergencies; when they do become concerned, there is no readily available data to guide program planning. 

## 5. Conclusions 

This study highlights the need to improve public health surveillance of backcountry travel emergencies and demonstrates a relatively quick, novel and inexpensive approach. Further work involving multiple sources of data can identify priority areas to strengthen emergency preparedness, integrate institutional and local knowledge [4], and bring attention to the need for community-focused measures and response capacity. 

## Abbreviations

The following abbreviations are used in this manuscript:

NWT: Northwest Territories
NU: Nunavut
SAR: Search and Rescue
